# HIV-1 molecular epidemiology among newly diagnosed HIV-1 individuals in Hebei, a low HIV prevalence province in China

**DOI:** 10.1371/journal.pone.0171481

**Published:** 2017-02-08

**Authors:** Xinli Lu, Xianjiang Kang, Yongjian Liu, Ze Cui, Wei Guo, Cuiying Zhao, Yan Li, Suliang Chen, Jingyun Li, Yuqi Zhang, Hongru Zhao

**Affiliations:** 1 College of Life Science, Hebei University, Baoding, Hebei, China; 2 Hebei Provincial Center for Disease Control and Prevention, Shjiazhuang, Hebei, China; 3 Department of AIDS Research, State Key Laboratory of Pathogen and Biosecurity, Beijing Institute of Microbiology and Epidemiology, Beijing, China; 4 NO. 201 hospital of the People’s Liberation Army of China, Liaoyang, Liaoning, China; National and Kapodistrian University of Athens, GREECE

## Abstract

New human immunodeficiency virus type 1 (HIV-1) diagnoses are increasing rapidly in Hebei. The aim of this study presents the most extensive HIV-1 molecular epidemiology investigation in Hebei province in China thus far. We have carried out the most extensive systematic cross-sectional study based on newly diagnosed HIV-1 positive individuals in 2013, and characterized the molecular epidemiology of HIV-1 based on full length gag-partial *pol* gene sequences in the whole of Hebei. Nine HIV-1 genotypes based on full length gag-partial *pol* gene sequence were identified among 610 newly diagnosed naïve individuals. The four main genotypes were circulating recombinant form (CRF)01_AE (53.4%), CRF07_BC (23.4%), subtype B (15.9%), and unique recombinant forms URFs (4.9%). Within 1 year, three new genotypes (subtype A1, CRF55_01B, CRF65_cpx), unknown before in Hebei, were first found among men who have sex with men (MSM). All nine genotypes were identified in the sexually contracted HIV-1 population. Among 30 URFs, six recombinant patterns were revealed, including CRF01_AE/BC (40.0%), CRF01_AE/B (23.3%), B/C (16.7%), CRF01_AE/C (13.3%), CRF01_AE/B/A2 (3.3%) and CRF01_AE/BC/A2 (3.3%), plus two potential CRFs. This study elucidated the complicated characteristics of HIV-1 molecular epidemiology in a low HIV-1 prevalence northern province of China and revealed the high level of HIV-1 genetic diversity. All nine HIV-1 genotypes circulating in Hebei have spread out of their initial risk groups into the general population through sexual contact, especially through MSM. This highlights the urgency of HIV prevention and control in China.

## Introduction

A recent report revealed that 1920s Kinshasa was the epicentre of the early spread of human immunodeficiency virus type 1 (HIV-1), and HIV-1 then spread across the world via transport networks[1]. Global reports indicate that there are almost 75 million HIV infections in the world[2]. In China, the first acquired immunodeficiency syndrome (AIDS) patient (an Argentinean) was diagnosed in Bejing[3]in 1985 and the first native HIV-1-infected patient with haemophilia was diagnosed at almost the same time in Hangzhou[4], China.

For over 30 years, HIV-1 has evolved rapidly, with an increasing number of genotypes and novel recombinant forms. Recombination between different genotype gene fragments is one of the main factors responsible for HIV-1 genetic evolution. A series of novel recombinant strains have been identified by scientists from China, including circulating recombinant form (CRF)07_BC and CRF08_BC among intravenous drug users (IDUs)[5], CRF55 01B[6] and CRF59 01B[7] among men who have sex with men (MSM), CRF61_BC[8], CRF57_BC[9], CRF62_BC[10], CRF64_BC[11] and CRF65_cpx[12] among IDUs and/or heterosexuals, and substantially different unique recombinant forms (URFs) among various at risk populations, enriching the global HIV-1 genetic data. Up to now, there are at least 12 HIV-1 genotypes in China[13]. Moreover, the predominant drivers of HIV-1 prevalence in China have obviously shifted, and heterosexual transmission has become the most common route[14]. However, the highest HIV-1 prevalence among MSM was found in newly diagnosed HIV-1 individuals in some provinces such as Liaoning, Beijing and Henan[15,16].

Hebei is a northern province of China, with 11 prefectures, and the gateway to Beijing, and neighbouring Henan in the south. Between 2005 and 2013, newly diagnosed HIV-1 individuals increased rapidly in Hebei, from 244 cases to 978 cases. Our recent study revealed that there were six HIV-1 genotypes circulating in Hebei in 2011, and subtype B, CRF01_AE and CRF07_BC were the most common genotypes[17]. In Hebei, the first recombinant form (CRF07_BC) was fund in 2002[18], followed by CRF01_AE and CRF08_BC in 2008[19], and URFs in 2011[17]. Among the newly diagnosed HIV-1 individuals, the proportion of MSM increased from 4.9% in 2005 to 62.2% in 2013[20], which is markedly higher than the data reported in 61 other cities in China[15]. Thus, MSM play a critical role in this increasing trend of HIV-1 prevalence in Hebei. The recombinant strains are arising frequently, especially in populations with multiple circulating HIV-1 genotypes[21].

In this study, we have carried out the most extensive systematic cross-sectional study based on newly diagnosed HIV-1 positive individuals in 2013, and characterized the molecular epidemiology of HIV-1 based on full length *gag*-partial *pol* gene sequences in the whole of Hebei. Hebei may not be representative of China, however,it can reflect HIV epidemic trend of the low HIV prevalence province, especially the provinces with the highest HIV-1 prevalence among MSM found in newly diagnosed HIV-1 individuals.

## Materials and methods

### Ethics statement

Written informed consent was obtained from all adult subjects and parents/guardians of HIV-1 positive minors/children enrolled in our study before blood collection. Our study was approved by the local Ethics Committee at Hebei Provincial Center for Disease Control and Prevention (CDC). All of the experimental methods were implemented in accordance with the approved regulations and guidelines, and the experimental protocols were approved by the institutional review boards of Hebei CDC.

### Study subjects

**Fig 1:**
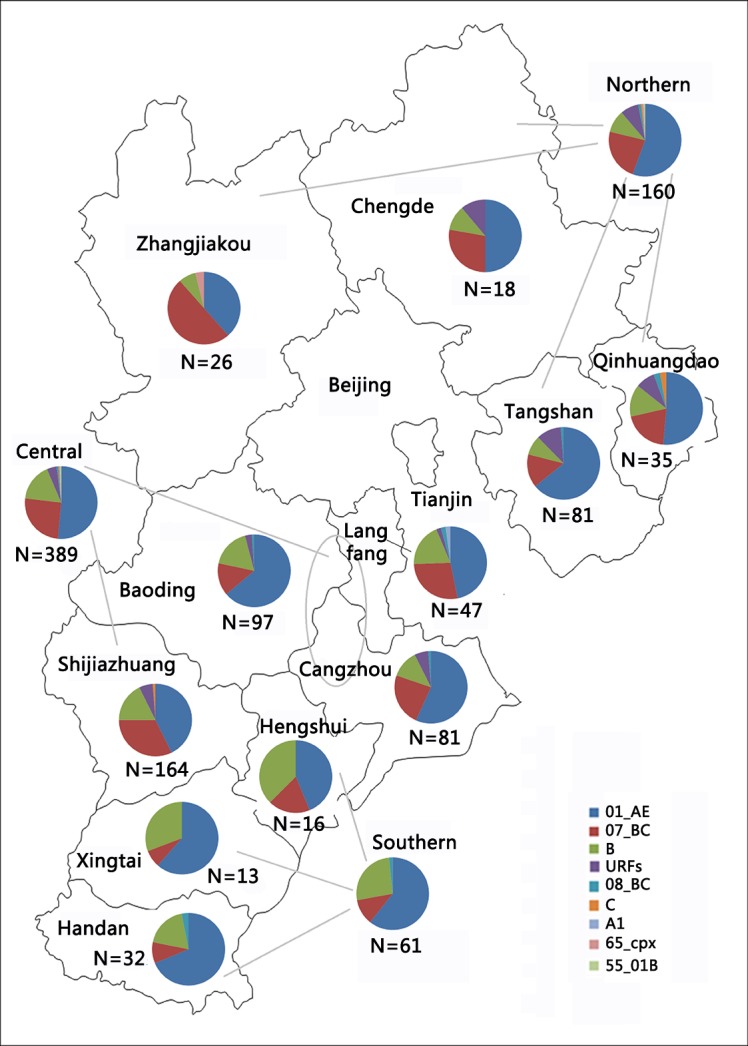
Geographic distribution of HIV-1 genotypes in Hebei in 2013. This figure is adapted from open access map: http://map.ps123.net/ditu/HTML/8939.html with Microsoft PowerPoint 2016.

This study presents the most extensive HIV-1 molecular epidemiology investigation in a province in China thus far. In 2013, a total of 978 individuals were newly diagnosed with HIV-1 infections and did not receive antiretroviral therapy (ART). Of these individuals, 50 recently infected MSM found at MSM sentinel surveillance points have been reported in our previous study[20]. In the present work, a total of 856 blood samples of the remaining 928 newly diagnosed individuals were collected from the 11 Hebei prefectures after obtaining written informed consent, accounting for 87.5% (856/978) of all HIV-1 infections. The 11 prefectures are grouped into three regions (Table 1) according to their proximity (Fig 1). HIV-1 infections were mainly concentrated in central (n = 520+43[20]), followed by northern (n = 216+7[20]), and southern (n = 120) in 2013.

**Table 1:** Demographic and epidemiological information of the study subjects.

Characteristics		Subjects	Subjects genotyped		χ^2^	p
			N	%		
**Gender**						
Male		741	536	72.3	0.539	0.463
Female		115	74	64.3		
**Age(years)**						
0–18		33	16	48.5	1.811	0.404
19–49		698	508	72.8		
50-		125	86	68.8		
**Marital status**						
Married		502	395	78.7	5.667	0.059
Unmarried		267	160	59.9		
Divorced/widowed		87	55	63.2		
**Ethnicity**						
Han		815	580	71.2	0.013	0.910
Minority		41	30 ^a^	73.2		
**Infection status**						
Previous infection		770	554	71.9	0.306	0.580
Recent infection		86	56	65.1		
**First CD4 counts (cells/μl)**						
≤200		347	269	77.5	2.527	0.283
201–500		443	290	65.5		
>500		66	51	77.3		
**Transmission routes**						
MSM		541	411	76.0	4.267	0.371
Heterosexual		278	172 ^b^	61.9		
Blood		6	2	33.3		
IDU		25	21^c^	84.0		
MTCT		6	4	66.7		
**Prefectures**						
Northern	Qinghuangdao	48	35	72.9	15.987	0.100
	Tangshan	94	81	86.2		
	Zhangjiakou	35	26	74.3		
	Chengde	39	18	46.2		
Central	Baoding	131	97	74.0		
	Cangzhou	99	81	81.8		
	Langfang	75	47	62.7		
	Shijiazhuang	215	164	76.3		
Southern	Handan	41	32	78.0		
	Xingtai	41	13	31.7		
	Hengshui	38	16	42.1		

MSM: Men who have sex with men; IDU: Intravenous drug user; MTCT: Mother-to-child transmission

^a^ The minorities included Yi (17 cases), Hui (6 cases), Man (5 cases) and Uyghur (2 cases).

^b^ Seven females were infected with HIV-1 through husband-to-wife heterosexual contact.

^c^ Four of 21 IDUs also had unprotected heterosexual contact.

Participants’ demographic data were obtained by face-to-face interviews using a standard questionnaire when we collected their blood samples.500 μl plasma separated from whole blood within 6 hours of collection was used to obtain HIV-1 nucleotide sequences for subsequent analysis.

### Extraction, purification and sequencing of HIV-1 RNA

Following previously reported methods[22, 23], HIV-1 RNA was extracted from 500 μl of plasma using high pure viral RNA kit (Qiagen, Valencia, CA, USA) and followed by full length *gag*(HXB2:763–2400) and *pol*(HXB2:2147–3462) genes amplification for HIV-1 genotyping. The PCR products were analysed using 1% agarose gel electrophoresis. Finally, PCR products separated by agarose gel electrophoresis were sequenced by Biomed (Beijing, China).

### HIV-1 sequence analysis

Initially, HIV-1 sequences were analysed and classified as pure subtypes, CRFs-like or URFs using the online REGA HIV-1 Subtyping Tool, version 2.0[24]. Initial genotypes were then confirmed using the neighbour-joining (N-J) phylogenetic tree and recombinant breakpoints analysis. All reference sequences (A–D, F–H, J, K, O, CRF01_AE, all CRFs associated with B/C, 01/BC and 01/B recombination) were obtained from Los Alamos HIV Sequence Database (http://www.hiv.lanl.gov/content/index). The original gene fragments sequenced successfully were edited and assembled as described previously[23]. Full length *gag* and partial *pol* gene sequences derived from the same subject were assembled together. The N-J phylogenetic tree was constructed using MEGA 5.0 with 1000 bootstrap replicates, based on Kimura 2-parameter Model. The analysis of recombinant breakpoints were implemented using the online jpHMM-HIV (http://jphmm.gobics.de/submission_hiv.html) and online RIP 3.0 (http://www.hiv.lanl.gov/content/sequence/RIP/RIP.html). Ambiguous recombinant forms analysed by the above methods were further confirmed using simplot 3.5.1 software with window size = 300 bp. The maximum-likelihood (ML) tree analysis wasperformed using MEGA 6.0 with 1,000 replicates. Viral *pol* gene sequences were submitted to the Stanford HIV Database (http://hivdb.stanford.edu/pages/algs/HIVdb.html), and then compared to gene sequence of a subtype B reference strain(HXB2). Drug resistance mutations and antiretroviral susceptibility were evaluated using the stanford DR algorithm.

### Statistical analysis

Statistical analyses for this study were performed using SPSS version 21.0 (SPSS Inc., Chicago, IL, USA). Mean or frequencies of demographic data (age, CD4 cell counts) were calculated. Categorical variables were analysed using the chi-square test. When more than 20% of cells had an expected count less than 5, the Fisher`s exact test was used. All tests were 2-sided, and a result was considered statistically significant when the *P*-value was less than 0.05.

## Results

### Demographic and epidemiologic information of subjects

As summarized in Table 1, 610 subjects were successfully genotyped based on the full length *gag*-partial *pol* (1.3 kb) gene sequences, achieving a positive rate of 71.3% (610/856). The sequence positive rate in eight of 11 prefectures was more than 60.0%, from 62.7% in Langfang to 86.2% in Tangshan. The constituent ratios of 610 genotyped subjects and the total of 856 newly diagnosed HIV-1-infected individuals showed no statistically significant differences by gender, age, marital status, ethnicity, transmission routes, infection status, first CD4 counts and prefectures. Therefore, the 610 genotyped subjects can completely represent HIV-1 epidemic strains circulating in the whole of Hebei province in 2013.

In this study, males accounted for 87.9% of the population (536/610). The mean age and the mean first CD4 counts of the 610 subjects were 35.6 (2–86) years and 245.1 (1–949) cells/μl, respectively. 64.8% (395/610) were married or cohabited, 28.2% (160/610) were unmarried and 9.0% (55/610) were divorced or widowed. According to ethnicity, 95.1% (580/610) of the study population were of Han ethnicity, and minorities included Yi (2.8%, 17/610), Hui (0.98%, 6/610), Man (0.8%, 5/610) and Uyghur (0.3%, 2/610). Recently infected subjects accounted for 9.2% (56/610). Sexual transmission accounted for 95.6% (583/610), including MSM (67.4%, 411/610) and heterosexual (28.2%, 172/610) transmission, followed by IDU (3.4%, 21/610), mother-to-child transmission (MTCT) (0.7%, 4/610) and blood transmission (0.3%, 2/610). Additionally, seven females of 172 heterosexuals were infected with HIV-1 through husband-to-wife heterosexual contact. Four of 21 IDUs also had unprotected heterosexual contact.

Furthermore, S1 Table shows that the distribution of transmission routes in northern, central and southern regions of Hebei were significantly different (χ^2^ = 7.598, *p* = 0.022). The proportion of MSM in all 11 prefectures except Xingtai (46.2%) exceeded 50.0%. Indeed, MSM in Tangshan, Baoding and Hengshui accounted for 88.9%, 81.4% and 81.3% of the study population, respectively. One hundred per cent of the subjects from Tangshan, Cangzhou, Chengde, Handan and Hengshui were only infected through MSM and heterosexual sex (S1 Table).

### HIV-1 genotype analysis

As shown in Table 2, from the 610 subjects successfully genotyped, a total of nine HIV-1 genotypes were identified, including CRF01_AE (53.4%, 326/610), CRF07_BC (23.4%, 143/610), subtype B (15.9%, 97/610), URFs (4.9%, 30/610), CRF08_BC (1.0%, 6/610), subtype C (0.5%, 3/610), CRF55_01B (0.5%, 3/610), subtype A1 (0.2%, 1/610) and CRF65_cpx (0.2%, 1/610). CRF01_AE, CRF07_BC and subtype B were the three main genotypes, accounting for 92.8% (566/610) of all subjects genotyped. Notably, the frequency of URFs (4.9%) was greater than that of CRF08_BC (1.0%) and ranked the fourth frequent genotype. In 2013, subtype A1, CRF55_01B and CRF65_cpx circulating in China were found for the first time in Hebei.

**Table 2:** Demographic and geographic distribution of HIV-1 genotypes in Hebei.

Characteristic		Subjects (%)	01_AE	07_BC	B	URFs	08_BC	C	A1	65_cpx	55_01B	χ^2^	p
**Total**		610 (100)	326 (53.4)	143 (23.4)	97 (15.9)	30 (4.9)	6 (1.0)	3 (0.5)	1 (0.2)	1 (0.2)	3 (0.5)		
**Gender**													
Male		536 (100)	290 (54.1)	129 (24.1)	80 (14.9)	28 (5.2)	3 (0.6)	1 (0.2)	1 (0.2)	1 (0.2)	3 (0.6)	17.360	0.018
Female		74 (100)	36 (48.6)	14 (18.9)	17 (23.0)	2 (2.7)	3 (4.1)	2 (2.7)	0 (0.0)	0 (0.0)	0 (0.0)		
**Age**													
≤14		3 (100)	3 (100.0)	0 (0.0)	0 (0.0)	0 (0.0)	0 (0.0)	0 (0.0)	0 (0.0)	0 (0.0)	0 (0.0)	8.801	0.456
15–24		131 (100)	73 (55.7)	31 (23.7)	20 (15.3)	6 (4.6)	1 (0.8)	0 (0.0)	0 (0.0)	0 (0.0)	0 (0.0)		
25–49		390 (100)	208 (53.3)	95 (24.4)	56 (14.4)	20 (5.1)	4 (1.0)	3 (0.8)	1 (0.3)	1 (0.3)	2 (0.5)		
≥50		86 (100)	42 (48.8)	17 (19.8)	21 (24.4)	4 (4.7)	1 (1.2)	0 (0.0)	0 (0.0)	0 (0.0)	1 (1.2)		
**First CD4 counts (cells/μl)**													
≤200		269 (100)	140 (52.0)	56 (20.8)	49 (18.2)	15 (5.6)	5 (1.9)	1 (0.4)	1 (0.4)	1 (0.4)	1 (0.4)	4.345	0.630
201–500		290 (100)	158 (54.5)	76 (26.2)	40 (13.8)	12 (4.1)	1 (0.3)	1 (0.3)	0 (0.0)	0 (0.0)	2 (0.7)		
>500		51 (100)	28 (54.9)	11 (21.6)	8 (15.7)	3 (5.9)	0 (0.0)	1 (2.0)	0 (0.0)	0 (0.0)	0 (0.0)		
**Transmission routes**													
MSM		411 (100)	234 (56.9)	92 (22.4)	58 (14.1)	21 (5.1)	2 (0.5)	0 (0.0)	1 (0.2)	1 (0.2)	2 (0.5)	46.587	<0.001^a^
Heterosexual		172 (100)	86 (50.0)	34 (19.8)	37 (21.5)	7 (4.1)	4 (2.3)	3 (1.7)	0 (0.0)	0 (0.0)	1 (0.6)	16.365	0.017^b^
MTCT		4 (100)	4 (100.0)	0 (0.0)	0 (0.0)	0 (0.0)	0 (0.0)	0 (0.0)	0 (0.0)	0 (0.0)	0 (0.0)		
IDU		21 (100)	2 (9.5)	17 (81.0)	0 (0.0)	2 (9.5)	0 (0.0)	0 (0.0)	0 (0.0)	0 (0.0)	0 (0.0)		
Blood		2 (100)	0 (0.0)	0 (0.0)	2 (100.0)	0 (0.0)	0 (0.0)	0 (0.0)	0 (0.0)	0 (0.0)	0 (0.0)		
**Marital Status**													
Married		395 (100)	211 (53.4)	89 (22.5)	61 (15.4)	25 (6.3)	4 (1.0)	1 (0.3)	1 (0.3)	1 (0.3)	2 (0.5)	8.522	0.202
Unmarried		160 (100)	86 (53.8)	36 (22.5)	30 (18.8)	4 (2.5)	1 (0.6)	2 (1.3)	0 (0.0)	0 (0.0)	1 (0.6)		
Divorced/Widowed		55 (100)	29 (52.7)	18 (32.7)	6 (10.9)	1 (1.8)	1 (1.8)	0 (0.0)	0 (0.0)	0 (0.0)	0 (0.0)		
**Ethnicity**													
Han		580 (100)	317 (54.7)	126 (21.7)	96 (16.6)	28 (4.8)	6 (1.0)	2 (0.3)	1 (0.2)	1 (0.2)	3 (0.5)	25.957	<0.001
Minority		30 (100) ^a^	9 (0.3)	17 (56.7)	1 (3.3)	2 (6.7)	0 (0.0)	1 (3.3)	0 (0.0)	0 (0.0)	0 (0.0)		
**Infection status**													
Previous infection		554 (100)	297 (53.6)	127 (22.9)	93 (16.8)	24 (4.3)	6 (1.1)	3 (0.5)	1 (0.2)	0 (0.0)	3 (0.5)	14.274	0.066
Recent infection		56 (100)	29 (51.8)	16 (28.6)	4 (7.1)	6 (10.7)	0 (0.0)	0 (0.0)	0 (0.0)	1 (1.8)	0 (0.0)		
**Prefectures**													
Northern	QHD	35 (100)	18 (51.4)	7 (20.0)	5 (14.3)	3 (8.6)	1 (2.9)	1 (2.9)	0 (0.0)	0 (0.0)	0 (0.0)	19.742	0.003^c^
	TS	81 (100)	52 (64.2)	12 (14.8)	7 (8.6)	8 (9.9)	1 (1.2)	0 (0.0)	0 (0.0)	0 (0.0)	1 (1.2)	6.924	0.031^d^
	ZJK	26 (100)	10 (38.5)	13 (50.0)	2 (7.7)	0 (0.0)	0 (0.0)	0 (0.0)	0 (0.0)	1 (3.8)	0 (0.0)		
	CD	18 (100)	9 (50.0)	5 (27.8)	2 (11.1)	2 (11.1)	0 (0.0)	0 (0.0)	0 (0.0)	0 (0.0)	0 (0.0)		
	Subtotal	160 (100)	89 (55.6)	37 (23.1)	16 (10.0)	13 (8.1)	2 (1.3)	1 (0.6)	0 (0.0)	1 (0.6)	1 (0.6)		
Central	BD	97 (100)	62 (63.9)	14 (14.4)	17 (17.5)	3 (3.1)	1 (1.0)	0 (0.0)	0 (0.0)	0 (0.0)	0 (0.0)		
	CZ	81 (100)	46 (56.8)	19 (23.5)	10 (12.3)	3 (3.7)	1 (1.2)	0 (0.0)	0 (0.0)	0 (0.0)	2 (2.5)		
	LF	47 (100)	22 (46.8)	13 (27.7)	9 (19.1)	1 (2.1)	1 (2.1)	0 (0.0)	1 (2.1)	0 (0.0)	0 (0.0)		
	SJ	164 (100)	70 (42.7)	53 (32.3)	29 (17.7)	10 (6.1)	0 (0.0)	2 (1.2)	0 (0.0)	0 (0.0)	0 (0.0)		
	Subtotal	389 (100)	200 (51.4)	99 (25.4)	65 (16.7)	17 (4.4)	3 (0.8)	2 (0.5)	1 (0.3)	0 (0.0)	2 (0.5)		
Southern	HD	32 (100)	22 (68.8)	3 (9.4)	6 (18.8)	0 (0.0)	1 (3.1)	0 (0.0)	0 (0.0)	0 (0.0)	0 (0.0)		
	XT	13 (100)	8 (61.5)	1 (7.7)	4 (30.8)	0 (0.0)	0 (0.0)	0 (0.0)	0 (0.0)	0 (0.0)	0 (0.0)		
	HS	16 (100)	7 (43.8)	3 (18.8)	6 (37.5)	0 (0.0)	0 (0.0)	0 (0.0)	0 (0.0)	0 (0.0)	0 (0.0)		
	Subtotal	61 (100)	37 (60.7)	7 (11.8)	16 (26.2)	0 (0.0)	1 (1.6)	0 (0.0)	0 (0.0)	0 (0.0)	0 (0.0)		

^a^ Compared HIV-1 genotype distribution among sexual contact, MTCT, IDU and blood transmission.

^b^ Compared HIV-1 genotype distribution between heterosexual and MSM sexual transmission.

^c^Compared HIV-1 genotype distribution in the northern, central and southern regions.

^d^ Compared HIV-1 URFs distribution in the northern, central and southern regions.

SJ: Shijiazhuang; TS: Tangshan; HD: Handan; BD: Baoding; XT: Xingtai; CZ: Cangzhou; CD: Chengde; HS: Hengshui; ZJK: Zhangjiakou; QHD: Qinghuangdao; LF: Langfang.

### Demographic distribution of HIV-1 genotypes

Table 2 reveals the epidemiological characteristics of HIV-1 genotype distribution. All nine genotypes were found in subjects with the following epidemiologic characteristics: male, 25–49 years old, first CD4 counts ≤ 200 cells/μl, married and Han, suggesting that male youth with Han ancestry were the major drivers of HIV-1 spread in Hebei. The main HIV-1 genotype distribution by gender, transmission routes and ethnicity showed significant statistical differences. Three main genotypes were distributed throughout almost all demographic characteristics, except for no CRF01_AE in blood transmission, no CRF07_BC in blood transmission, MTCT and subjects ≤ 14 years of age, and no subtype B in MTCT, IDUs and subjects ≤ 14 years of age. With the exception of CRF07_BC and subtype B being the most frequent genotypes in IDUs (81.0%) and minorities (56.7%), and in blood transmission (100.0%), respectively, the prevalence of CRF01_AE in nearly all demographic characteristics was the highest, exceeding 50.0%.

**Fig 2:**
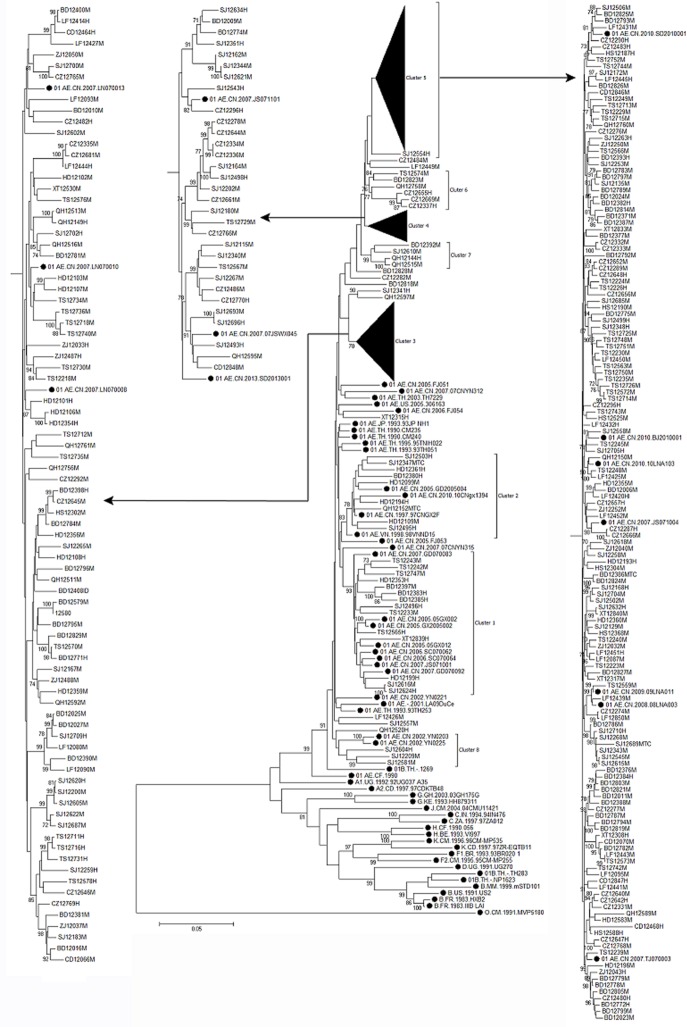
Phylogenetic tree analysis based on HIV-1 full length *gag*-partial *pol* gene sequences from CRF01_AE strains identifies eight distinct CRF01_AE Clusters. The maximum-likelihood tree was constructed using MEGA 6.0 with 1000 bootstrap replicates. The referencesequences (A–D, F–H, J, K, O, CRF01_AE) were obtained from the HIV database (http://www.hiv.lanl.gov/content/index). Bootstrap values ≥70% are shown in the tree. The scale length indicates 5% nucleotide sequence divergence. Black dot denotes the reference sequences.The left capital of sample ID denotes prefecture: HS, Hengshui; HD, Handan; XT, Xingtai; SJ, Shijiazhuang; BD, Baoding; CZ, Cangzhou; LF, Langfang; TS, Tangshan; CD, Chengde; ZJ, Zhangjiakou; QH, Qinhuangdao. The right capital of sample ID denotes transmission route: M, MSM; H, Heterosexual; MTC, Mother to child; ID, Intravenous drug. Within the reference sequence ID, LN, YN, JS, GD, SC, SD, TJ, BJ and GX(gx) denote Liaoning, Yunnan, Jiangsu, Guangdong, Sichuan, Shandong, Tianjin, Beijing and Guangxi, repectively.

All nine genotypes were identified in the population who sexually contracted HIV-1, including eight genotypes (except subtype C) in MSM and seven genotypes (except subtype A and CRF65_cpx) in heterosexuals. Moreover, HIV-1 genotype distribution between heterosexual and MSM sexual transmission showed the significant difference (χ^2^ = 16.365, *p* = 0.017). For example, in the ML tree analysis based on full-length *gag*-partial *pol* (1.3kb) sequences (Fig 2), eight clusters designated clusters 1–8 were observed among CRF01_AE sequences. Each cluster contained more than 3 our sequences with high branch supports. Clusters 3–8 indicated that the number of sequences was far more from MSM than other risk groups, which suggests that the CRF01_AE genotype is far more prevalent in MSM than other risk groups. However, in cluster 1 and cluster 2, the number of sequences was far more from other risk groups than MSM, and our sequences closely clustered together with those from Guangxi, Guangdong and Sichuan. Close relationship between our sequences with those from Liaoning (cluster 3), Jiangsu (cluster 4), Yunnan (cluster 8) and neighbouring provinces (Shandong, Tianjin, Beijing, etc.) of Hebei (cluster 5) were also observed in the ML tree. Moreover, cluster 6 and cluster 7 provide a strong evidence that HIV-1 CRF01_AE strains are circulating in different prefectures of Hebei through sexual transmission, especially MSM.

Only one genotype was identified in MTCT (CRF01_AE) and blood transmission (subtype B), respectively. For infection status, eight of nine genotypes were found in previously infected subjects, noticeably more than the five genotypes found in recently infected subjects. One of three subjects with genotypes found for the first time in this study was recently infected with CRF65_cpx. URFs were found in all demographic groups except MTCT and blood transmission, and differentially distributed in different infection status (*p* = 0.048). 93.3% (28/30) of URFs were found in subjects with sexually contracted disease, including MSM (4.8%, 21/411) and heterosexuals (4.1%, 7/172). 6.7% (2/30) of URFs were identified in two IDUs, and one of the two IDUs had not only injected drugs but also had risky heterosexual sexual behaviours. The prevalence of HIV-1 genotypes in different demographic groups is indicated in Table 2.

### Geographic distribution of HIV-1 genotypes

As indicated in Fig 1 and Table 2, the three main genotypes (CRF01_AE, CRF07_BC and subtype B) were extensively distributed in all 11 prefectures and showed significant differences (χ^2^ = 45.555, *p* = 0.001). In Zhangjiakou, the prevalence of CRF07_BC (50.0%) was higher than that of CRF01_AE (38.5%), being the most frequent genotype. However, in the remaining 10 prefectures, CRF01_AE was the most frequent, and the prevalence of CRF01_AE exceeded 50% in eight of the 10 prefectures, notably up to 68.8% in Handan. Additionally, the occurrence odds of URFs in northern, central and southern regions of Hebei were unequal (χ^2^ = 6.924, *p*< 0.031). The prevalence of URFs exceeded 10% only in Chengde, and the prevalence of URFs in the remaining 10 prefectures is shown in Table 2.

In the northern region, MSM (76.9%) and heterosexuals (20.6%) were the two main risk groups. IDUs and MTCT only accounted for 1.9% and 0.6%, respectively (S1 Table). Eight of 9 identified genotypes (except subtype A1) were found here (Fig 1 and Table 2). CRF01_AE (55.6%) was the most prevalent, followed by CRF07_BC (23.1%), subtype B (10.0%), URFs (8.1%). In this region, two new CRFs (CRF65_cpx, 0.6%; CRF55_01B, 0.6%) were found circulating in China for the first time (Table 2).

In the central region, there were four risk groups, including MSM (63.2%), heterosexuals (31.4%), IDUs (4.6%) and MTCT (0.8%) (S1 Table). Eight genotypes were identified. CRF01_AE (51.4%), CRF07_BC (25.4%), subtype B (16.7%) and URFs (4.4%) were the four main genotypes. The prevalence of the four other minor genotypes was less than 1.0%, including CRF08_BC (0.8%), subtype C (0.5%), subtype A1 (0.3%) and CRF55_01B (0.5%). In this region, subtype A1 and CRF55_01B were first found circulating in China (Table 2). The distribution of prevalent HIV-1 strains in northern and central regions showed no significant differences (χ^2^ = 7.032, *p* = 0.070).

In the southern region, there were three risk groups. MSM (68.9%) and heterosexuals (27.9%) accounted for 96.7% of all subjects. Notably, blood transmission (3.3%) was present solely in this region (S1 Table). There were only two subjects infected with HIV-1 through blood transfusion. One was female farmer aged 42 years and the other was male farmer aged 47 years. These two cases who had the history of blood transfusion in the 1990s[19] were from two unlinked counties of Xingtai, respectively.Only four genotypes were found here. CRF01_AE (60.7%) was the most dominant genotype, followed by subtype B (26.2%), CRF07_BC (11.8%) and CRF08_BC (1.6%). No URFs were found. The prevalence of HIV-1 genotypes in this region was significantly different from the northern and central regions (Table 2).

### Identification of URFs

**Fig 3:**
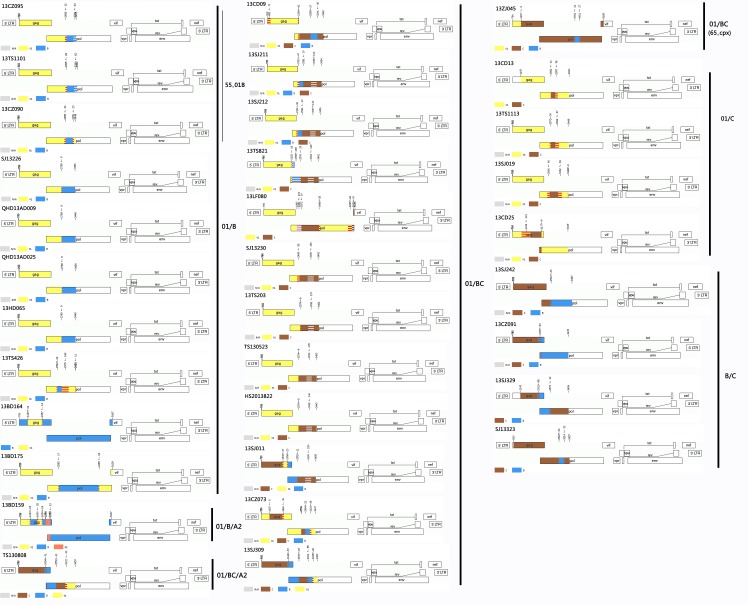
Genomic mosaic structure maps of 34 recombinant forms unknown before in Hebei. The jpHMM-HIV Program (http://jphmm.gobics.de/submission_hiv.html) was used to generate the mosaic structure maps based on HXB2 numbering. The different coloured symbols represent different genotype gene fragments. N/A denotes the missing information due to uninformative genotype models. 13CZ026 (underlined) listed in S3 Fig which was subtype C identified using jpHMM-HIV is not shown.

S1 Fig indicates that a total of 34 recombinant forms, which were unknown before in Hebei, were identified in this study. Information on 34 subjects harbouring HIV-1 recombinant strains is presented in Table 3. Of these recombinant forms, with the exception of two IDUs (one subject with intravenous drug use and unprotected heterosexual contact), all of the remaining subjects with these recombinant strains were infected through sexual contact (eight heterosexuals, 24 MSM). The analyses of recombinant breakpoints and the neighbour-joining (N-J) tree (S1 Fig) revealed that 13CZ095, 13TS1101 and 13CZ090 in cluster 1 (S1 and S2 Figs) and 13ZJ045 (S1 and S3 Figs) were diagnosed asCRF55_01B and CRF65_cpx, respectively. The remaining 30 sequences did not cluster with pure subtypes or CRFs reference sequences (S1–S3 Figs), and the breakpoints analysis also confirmed they were URFs (Fig 3).

**Table 3:** Information on subjects harbouring HIV-1 recombinant strains unknown before in Hebei, China.

Sample ID	Recombinant pattern	Prefecture	Gender	Age	Nationality	Marital status	BED	Transmission route	Collection date	First CD4 count
13BD159	CRF01_AE/B/A2	BD	Male	70	Han	Married		MSM	10-Oct.-13	110
13BD164	CRF01_AE/B	BD	Male	25	Han	Married	RECENT	MSM	15-Oct.-13	17
13BD175	CRF01_AE/B	BD	Male	32	Han	Married	RECENT	MSM	4-Nov.-13	104
13ZJ045	CRF01_AE/BC	ZJK	Male	32	Han	Married	RECENT	MSM	11-Nov.-13	27
13LF080	CRF01_AE/C	LF	Male	29	Han	Married		MSM	22-Ju.-13	60
13SJ011	CRF01_AE/BC	SJ	Male	48	Han	Widowed		Heterosexual	21-Jan.-13	260
13SJ019	CRF01_AE/C	SJ	Male	30	Han	Unmarried		IDU	31-Jan.-13	199
QHD13AD009	CRF01_AE/B	QHD	Male	42	Han	Married		Heterosexual	19-Feb.-13	131
13TS203	CRF01_AE/BC	TS	Male	20	Han	Unmarried		MSM	21-Feb.-13	559
13TS426	CRF01_AE/B	TS	Male	44	Han	Married		MSM	16-Apr.-13	266
13CZ026	B/C	CZ	Male	23	Han	Married	RECENT	Heterosexual	1-Apr.-13	16
13CD09	CRF01_AE/BC	CD	Male	21	Man	Unmarried		MSM	20-Mar.-13	331
13CD13	CRF01_AE/C	CD	Male	29	Man	Married		MSM	22-Apr.-13	325
13CD25	CRF01_AE/C	CD	Male	24	Han	Married		MSM	6-Jun-13	342
QHD13AD025	CRF01_AE/B	QHD	Male	52	Han	Married		MSM	1-Apr.-13	113
13SJ211	CRF01_AE/BC	SJ	Male	29	Han	Married	RECENT	MSM	1-Aug.-13	194
13SJ212	CRF01_AE/BC	SJ	Male	59	Han	Married	RECENT	Heterosexual	9-Aug.-13	255
SJ13226	CRF01_AE/B	SJ	Male	32	Han	Married	RECENT	MSM	19-Aug.-13	556
SJ13230	CRF01_AE/BC	SJ	Male	40	Han	Married		MSM	20-Aug.-13	239
TS130523	CRF01_AE/BC	TS	Male	30	Han	Married		MSM	24-May-13	211
13SJ242	B/C	SJ	Male	34	Han	Married		MSM	10-Sep.-13	600
13SJ309	CRF01_AE/BC	SJ	Male	36	Han	Married		Heterosexual	12-Jan.-13	257
SJ13323	B/C	SJ(SC)	Male	27	Yi	Married		IDU/heterosexual	27-Nov-13	267
13CZ073	CRF01_AE/BC	CZ	Male	20	Han	Unmarried		MSM	24-Jan.-13	56
13CZ090	CRF01_AE/B	CZ	Male	35	Han	Married		Heterosexual	21-Aug.-13	326
13CZ091^a^	B/C	CZ	Female	21	Han	Married		Heterosexual	21-Aug.-13	233
13CZ095	CRF01_AE/B	CZ	Male	50	Han	Married		MSM	26-Aug.-13	345
13SJ329	B/C	SJ	Female	45	Han	Married		Heterosexual	28-Nov.-13	232
TS130808	BC/CRF01_AE/A2	TS	Male	48	Han	Married	RECENT	MSM	15-Aug-13	201
13TS821	CRF01_AE/BC	TS	Male	21	Han	Married		MSM	29-Aug-13	32
HS2013822	CRF01_AE/BC	HS	Male	30	Han	Married		MSM	29-Aug.-13	37
13TS1101	CRF01_AE/B	TS	Male	25	Han	Unmarried		MSM	8-Nov.-13	167
13TS1113	CRF01_AE/C	TS	Male	30	Han	Married		MSM	8-Nov.-13	353
13HD065	CRF01_AE/B	HD	Male	53	Han	Married		MSM	14-Nov.-13	171

^a^ Transmission between newly-married couples, she was infected with HIV-1 through sexual contact with her husband.

MSM: Men who have sex with men.

IDU: Intravenous drug user.

RECENT: recently infected individuals.

13ZJ045 was identified as CRF65_cpx; 13CZ090, 13CZ095 and 13TS1101 were identified as CRF55_01B.

Among 30 URF sequences, the breakpoints analysis (Fig 3) revealed that six recombinant patterns were confirmed using jpHMM-HIV and RIP 3.0. CRF01_AE/BC was the most common recombinant pattern, accounting for 40% (12/30), followed by CRF01_AE/B (23.3%, 7/30), B/C (16.7%, 5/30), CRF01_AE/C (13.3%, 4/30), CRF01_AE/B/A2 (3.3%, 1/30) and CRF01_AE/BC/A2 (3.3%, 1/30). In our present work, nearly all of genotype gene fragments (except subtype A2) composed of these URFs are circulating in Hebei. 83.3% (25/30) of URFs harboured CRF01_AE gene fragments, followed by BC (60.0%, 18/30), subtype B (26.7%, 8/30), subtype C (13.3%, 4/30), and subtype A2 (6.7%, 2/30). In the CRF01_AE/B pattern, there were two mosaic structures: one that was a subtype B fragment inserted into a CRF01_AE backbone (six sequences) in the *pol* gene region. The other sequence contained a CRF01_AE segment in the *gag* gene region within a subtype B backbone. In the CRF01_AE/BC pattern, nine sequences contained only CRF01_AE in *gag* region, and CRF01_AE gene fragment was combined with BC gene fragment in *pol* region. Among the remaining three CRF01_AE/BC sequences, two contained a small CRF01_AE segment within a genotype BC backbone, respectively, and one contained a subtype C gene fragment in the *gag* region and a BC gene fragment in *pol* region within a CRF01_AE backbone, respectively. Of 12 CRF01_AE/BC sequences, the mosaic structure of sample 13CZ073 based on full length *gag*-partial *pol* was the most complicated(Fig 3).

In the CRF01_AE/C pattern, all four sequences harboured a genomic structure with a subtype C fragment within a CRF01_AE backbone, and the recombination positions located in the overlapping site between *gag* and *pol* region (one sequence) and *pol* region (three sequences), respectively. In the B/C pattern, the recombination positions of subtype B and subtype C located in the overlapping gene region (two sequences) of HIV-1 *gag* and *pol* genes, and *pol* region (three sequences), respectively. Only one sequence (13BD159) was identified as a CRF01_AE/B/A2 recombinant. Furthermore, five recombination positions accumulated in the *gag* region. However, the recombination positions of TS130808 within CRF01_AE/BC/A2 were mainly located in the *pol* region, and were previously described in detail by Lu et al[25]. Of these six recombinant forms identified, CRF01_AE/B, CRF01_AE/BC and B/C were found in 72 CRFs obtained from the HIV database (http://www.hiv.lanl.gov/content/sequence/HIV/CRFs/ CRFs.html).

Among 30 URFs, three clusters were identified from 13 sequences (S1 Fig). Cluster 2 and cluster 3 were supported by a 99% bootstrap value, respectively, distantly associated with all reference sequences. Cluster 2 and cluster 3 were potential CRFs (pCRFs) previously described in detail by Lu et al[25]. However, compared with three sequences in cluster 2 reported by Lu et al[25], four of our sequences were included in cluster 2 in this study, and 13TS203 obtained from Tangshan was a new sequence with identical mosaic structure (Fig 3). In cluster 4, the recombinant breakpoints of all five sequences obtained from Chengde (13CD09), Shijiazhuang (13SJ211 and 13SJ212), Tangshan (13TS821) and Langfang (13LF080) were located in the *pol* region. As shown in Fig 3, 13SJ211 and 13SJ212 contained identical genomic mosaic structures, however, the mosaic structures of 13CD09, 13TS821 and 13LF080 were different: 13CD09 and 13LF080 contained only subtype B gene fragments at positions 2842–3177 and 2462–2572 approximately, respectively. The mosaic structure of 13TS821 harboured a recombinant breakpoint at overlapping site of HIV-1 *gag* and *pol* gene sequences, different from the other four sequences. According to the criteria for identification of a new CRF[26], the above fact demonstrated that cluster 4 should not be a pCRF.

### Surveillance of TDR-related mutations

As shown in S2 Table, 17.2% (105/610) of 610 subjects with *pol* gene (1.3kb) harbored HIV-1 transmitted drug resistance (TDR)-related mutations, including 5.6% (34/610) for protease inhibitors (PIs) mutations, 2.3% (14/610) for nucleoside reverse transcriptase inhibitors (NRTIs) mutations, and 9.3% (57/610) for non-nucleoside reverse transcriptase inhibitors (NNRTIs) mutations. V179D/E/T was the most frequent mutation, accounting for 6.7%(41/610), followed by M46L(1.6%, 10/610), K20I (1.6%, 10/610), M46I (1.6%, 10/610), E138AE(0.7%, 4/610), T69N (0.7%, 4/610), K219E/Q/R (0.5%, 3/610), Q58E (0.5%, 3/610), K65EK (0.5%, 3/610), and other mutations(the prevalence of each mutation was ≤0.3%). Of these mutations, L10F, K20I, L33F, K65EK, T69N, V75L, K219E/Q/R and V179D/E/T conferred potential low-level resistance to antiretroviral drugs. The remaining mutations resulted in different-level resistance to antiretroviral drugs, from low-level resistance to high-level resistance.

TDR-related mutations were only found in the sexual transmission subjects. Furthermore, 64.8% (68/105) of subjects containing mutations were MSM and 35.2% (37/105) were heterosexuals. TDR-related mutations were found in seven of nine genotypes. Among subjects with TDR-related mutations, 69.5% (73/105) contained CRF01_AE, 15.2% (16/105) contained CRF07_BC, and 6.7% (7/105) contained subtype B. The percentages of TDR-related mutations in subtype A and CRF55_01B were 100.0% (1/1) and 100.0% (3/3), respectively, followed by 33.3% (2/6) in CRF08_BC, 22.4% (73/326) in CRF01_AE, 10.0% (3/30) in URFs, 11.1% (16/143) in CRF07_BC, and 7.2%(7/97) in subtype B.

## Discussion

By the end of 2013, a total of 4148 individuals were diagnosed with HIV-1 infections in Hebei, accounting for 0.9% of the HIV-infected individuals nationwide, thus Hebei is ranked 22^nd^ of China’s provinces[20]in terms of survival HIV or AIDS individuals. In contrast, HIV-infected individuals in Yunnan, Guangxi and Sichuan accounted for 45% of HIV-infected individuals nationwide (http://www.qianzhan.com/news/detail/365/141203-8a6e790b.html). Furthermore, it was estimated that the prevalence of HIV-1 in all populations was 0.011% in Hebei, significantly lower than 0.059% in China and 0.8% in the world (http://yanzhao.yzdsb.com.cn/system/2014/11/27/014005079.shtml), maintaining a low HIV-1 prevalence. In past years, some areas with high HIV prevalence, such as Yunnan, Sichuan and Guangxi, have received more attention, including policy and funding, than areas with a low HIV prevalence.

This study is the most extensive HIV-1 molecular epidemiological investigation in a province in China. Our present work revealed a high level of HIV-1 genetic diversity and the seriousness of the HIV epidemic in Hebei. A total of nine genotypes were identified among newly diagnosed HIV-1 positive individuals in 2013 in Hebei (Table 2). Compared with our report in 2011[17], a major shift of HIV-1 genotypes circulating in Hebei was revealed in this study, and CRF01_AE and CRF07_BC have replaced subtype B to become the most common and second common genotypein this area, respectively. Moreover, one subtype (subtype A1), 2 CRFs (CRF55_01B and CRF65_cpx) and 2 pCRFs were first identified within this year. These results suggest that Hebei has become one of China’s provinces[13]with the most HIV-1 genotypes.

HIV-1 diversity varied greatly in different demographic characteristics and different geographical regions. All nine genotypes were found in subjects with the following epidemiologic characteristics: male, 25–49 years old, CD4 counts ≤ 200 cells/μl, married and Han. Distribution of the three main genotypes showed significant statistical differences in gender, transmission routes and ethnicity. CRF01_AE predominated among nearly all demographic characteristics (more than 50.0%), with the exception of CRF07_BC and subtype B being the most frequent genotypes in IDUs (81.0%) and minorities (56.7%), and in blood transmission (100.0%), respectively. We found the highest level of viral diversity was among the group with sexually contracted HIV-1: all nine genotype categories were identified in this population, including eight genotypes in MSM and seven genotypes in heterosexuals. However, only one subtype was found in blood (subtype B) and MTCT (CRF01_AE), respectively. Furthermore, the distribution of HIV-1 genotypes in the northern, central and southern regions was significantly different. Eight genotypes and four risk groups were observed in the northern and central regions respectively, and CRF01_AE was the most frequent, followed by CRF07_BC, subtype B. However, four genotypes and three risk groups were found in the southern region, and subtype B replaced CRF07_BC as the second prevalent strain. Compared with the northern and central regions, HIV-1 genetic characteristics in the southern region with less HIV-1 infections were simpler. The above fact suggests that all nine HIV-1 genotypes circulating in Hebei have spread out of their initial risk groups into the general population through sexual contact, especially through MSM.

In previous reports[12, 14, 27], no CRF65_cpx were found in northern China. Our study is the first to confirm the emergence of this CRF in Hebei, a northern province of China. One subject with CRF65_cpx was recently infected through MSM and loved to travel around China, suggesting that the homosexual contact on his trip was the main factor behind CRF65_cpx being introduced into Hebei. Of three subjects with CRF55_01B, two subjects were infected through MSM, and another was infected through heterosexual contact.

The overall prevalence of URFs in Hebei increased rapidly within 3 years, from 1.4% in 2011[13]to 4.9% in 2013. Meanwhile, the recombinant patterns were more and more complicated, from only one (CRF01_AE/B) in 2011 to six patterns in 2013. Compared with the provinces with high HIV-1 prevalence, Hebei had a higher prevalence of URFs than Henan[28, 29]and Guangxi[29, 30], but lower than that in Yunnan[29, 31, 32]. Furthermore, HIV-1 recombination patterns in Hebei were more complicated than that in these three provinces. Additionally, 96.7% of URFs were associated with sexual contact, especially MSM (70.0%). We deduced that the geographic differences in the HIV-1 epidemic will change with time, and tend to decrease, especially in areas where the sexual transmission is the most frequent route of HIV spread.

As the major genotypes, the frequencies of CRF01_AE, BC and subtype B gene fragments recombined into URFs were also the highest, accounting for 83.3%, 60.0% and 26.7%, respectively, which provides new evidence for the opinion that the co-circulation of different HIV-1 genotypes results in the occurrence of novel recombination forms. Especially, two pCRFs originated from CRF01_AE and subtype B, and CRF01_AE and CRF07_BC respectively were also observed. As reported previously, HIV-1 genotype and viral tropism are the most important factors affecting the development of disease[33]. For example, the CRF01_AE strain has the strongest ability to infect people and cause disease, and is almost two times as efficient as the non-CRF01_AE strain[34, 35, 36]. In this study, in contrast to the most prevalent genotype (CRF07_BC, 35.5%) in China[13], the prevalence of CRF01_AE (53.4%) was the highest in Hebei, far above that of CRF07_BC (23.4%) and subtype B (15.9%). Moreover, the CRF01_AE strains from Hebei were closely related to southern provinces of China and neighbouring provinces of Hebei, and circulating in 11 prefectures of Hebei. Among 105 subjects with TDR-related mutations, 69.5% contained CRF01_AE. This hints that the virulence of HIV-1 CRF01_AE and recombinant strains circulating in Hebei obviously increases and significantly accelerates the progress of AIDS.

In contrast to the heterosexual transmission being the predominant route in China[13, 14], MSM transmission has become the most common transmission route in Hebei[20]. Some reports indicated that the recent HIV-1 infection rate among MSM (7.0–8.0%)[37, 38] was significantly higher than that among heterosexuals (0.5–4.4%)[39, 40, 41]. Hence, since the first HIV-1 MSM transmission was identified in 1997, MSM have attributed tremendously to the HIV-1 epidemic in Hebei. In this study, the high level of HIV-1 diversity and recombinant patterns were characterised in a population who had caught HIV through sexual contact. Particularly, HIV-1 diversity among MSM in Hebei was more complicated than that in most provinces of China[13]. Furthermore, HIV-1 resistant strains were only found in sexual contact population, especially MSM (64.8%). These results suggest that the HIV-1 epidemic among MSM constitutes the most predominant driver of the increased HIV-1 prevalence in Hebei. We must take effective measures to intervene in high risk sexual behaviours to control the increasing trend of HIV-1 prevalence, for example through increased education on sexually transmitted diseases and methods of protection.

There are two limitations in this study. First, we attempted to amplify and sequence HIV-1 *gag* and *pol* gene sequences from as many samples as possible. However, sequences were not successfully obtained from all the samples due to limited blood plasma volume, the poor quality of samples, the low viral load and limited interim storage conditions (during the sampling collection). Second, full length *gag*-partial *pol* sequences used for identification of HIV-1 genotypes maybe underestimate the prevalence of novel recombinant strains. Further studies will be very important to identify pCRFs among URFs basing on full length HIV-1 gene sequences.

## Conclusions

Our present study elucidated the complicated characteristics of HIV-1 molecular epidemiology in a low HIV-1 prevalence northern province of China and revealed the high level of HIV-1 genetic diversity. HIV-1 is evolving quickly and spreading through sexual exposure, especially through MSM in Hebei. Our findings highlight the urgency of HIV prevention and control in China, and suggest that the government should pay greater attention to the development of HIV-1 in the low HIV-1 prevalence areas. We should take great efforts to reduce diverse risky sexual behaviours to slow the spread of the epidemic.

## Supporting information

S1 Fig The N-J phylogenetic tree analysis based on HIV-1 full length *gag*-partial *pol* gene sequences from 34 recombinant forms unknown before in Hebei. The reference gene sequences (A–D, F–H, J, K, O, CRF01_AE) were obtained from the HIV database (http://www.hiv.lanl.gov/content/index). The N-J phylogenetic tree was constructed using MEGA 5.0 with 1000 bootstrap replicates, based on Kimura 2-parameter Model. Bootstrap values ≥70% are shown in the tree. The scale length indicates 2% nucleotide sequence divergence.
(TIF)

S2 Fig The N-J phylogenetic tree analysis associated with 01/B recombinant strains. (TIF)

S3 Fig The N-J phylogenetic tree analysis associated with 01/BC (CRF65_cpx) and B/C recombinant strains. Of B/C recombinant strains, although jpMM-HIV analysis indicated that 13CZ026 (underlined) was subtype C, the N-J tree (Fig 2, S3), SimPlot 3.5.1 and RIP 3.0 (window size = 300) analysis confirmed that this recombinant strain was composed of subtype B and subtype C, and a small subtype B gene fragment was inserted into a subtype C backbone in the *pol* region.
(TIF)

S1 Table Different risk groups in 11 prefectures of Hebei in 2013. MSM: men who have sex with men. IDU: intravenous drug user. MTCT: mother to child transmission.
(DOC)

S2 Table HIV-1 TDR-related mutations among 610 newly diagnosed-naïve individuals in 2013, Hebei. PIs:Protease inhibitors; NRTIs: Nucleoside reverse transcriptase inhibitors; NNRTIs: Non-nucleoside reverse transcriptase inhibitors.
(DOC)

## References

[1] Faria NR, Rambaut A, Suchard MA, Baele G, Bedford T, Ward MJ, et al HIV epidemiology. The early spread and epidemic ignition of HIV-1 in human populations. Science. 2014;346(6205):56–61. 10.1126/science.1256739 25278604 PMC4254776

[2] UNAIDS. Global Reports-UNAIDS report on the global AIDS epidemic 2013. UNAIDS; Geneva: 2013.

[3] Zheng YY. First case of AIDS diagnosed in China. NatI Med J Chin. 1988; 68(1): 5–6. 3133093

[4] Zeng Y, Fan J, Zhang Q, Wang PC, Tang DJ, Zhon SC, et a1 Detection of antibody to LAV/HTLV-III in sera from hemophiliacs in China. AIDS Res. 1986; 2 Suppl1:S147–149. 3103638

[5] Feng Y, Takebe Y, Wei H, He X, Hsi JH, Li Z, et al Geographic origin and evolutionary history of China's two predominant HIV-1 circulating recombinant forms, CRF07_BC and CRF08_BC. Sci Rep. 2016;6:19279 10.1038/srep19279 26763952 PMC4725877

[6] Han XX, An M, Zhang W, Cai W, Chen X, Takebe Y, et a1 Genome sequences of an novel HIV-1 circulating recombinant form, CRF55_01B, identified in China. Genome Announc. 2013; 1(1): e00050–12. 10.1128/genomeA.00050-12 23405298 PMC3569284

[7] Zhang WQ, Han X, An M, Zhao B, Hu Q, Chu Z, et a1 Identification and characterization of a novel HIV-l circulating recombinant form(CRF59_01B) identified among men-who-have-sex-with-men in China. PLoS One. 2014;9(6):e99693 10.1371/journal.pone.0099693 24978029 PMC4076182

[8] Li X, Ning C, He X, Yang Y, Li F, Xing H, et a1 Genome sequences of a novel HIV-1 circulating recombimnt form (CRF6l_BC) idemified among hetemsexuals in China. Genome Ailnounc. 2013; 1(3):e00326–13.

[9] Wei HM, Liu Y, Feng Y, Hsi J, Xing H, He X, et a1 Genome sequence of a novel HIV-l circulating recombinant form (CRF57_BC) identified from Yunnan, China. AIDS Res Hum Retroviruses. 2014; 30(4):384–388. 10.1089/AID.2013.0228 24205935 PMC3976592

[10] Wei HM, His J, Feng Y, Xing H, He X, Liao L, et a1 Identification of a novel HIV-1 circulating recombinant form (CRF62_BC) in western Yunnan of China. AIDS Res Hum Retmviruses. 2014; 30(4):380–3. 10.1089/aid.2013.0235 PMC3976572 24164474

[11] Hsi J, Wei H, Xing H, Feng Y, He X, Liao L, et a1 Genome sequence of a novel HIV-l circulating recombinant form (CRF64_BC) identified in Yunnan, China. AIDS Res Hum Retmviruses. 2014; 30(4):389–93. 10.1089/aid.2013.0234 PMC3976579 24205972

[12] Feng Y, Wei H, Hsi J, Xing H, He X, Liao L, et a1 Identification of a novel HIV type l circulating recombinant form(CRF65_cpx) composed of CRF0l_AE and subtypes B and C in Westem Yunnan, China. AIDS Res Hum Retroviruses. 2014; 30(6):598–602. 10.1089/AID.2013.0233 24279591 PMC4046203

[13] He X, Xing H, Ruan Y, Hong K, Cheng C, Hu Y, et al A Comprehensive mapping of HIV-1 genotypes in various risk groups and regions across China based on a nationwide molecular epidemiologic survey. PLoS One. 2012;7(10):e47289 10.1371/journal.pone.0047289 23056619 PMC3466245

[14] Wang N, Zhong P. Molecular epidemiology of HIV in China:1985–2015. Chin J Epidemiol. 2015; 36(6):541–6. 26564620

[15] Wu Z, Xu J, Liu E, Mao Y, Xiao Y, Sun X, et al HIV and syphilis prevalence among men who have sex with men:a cross-sectional survey of 61 cities in China. Clin Infect Dis. 2013; 57(2):298–309. 10.1093/cid/cit210 23580732 PMC3689345

[16] China, M. o.H.o.t.P.s.R.o. Estimates for the HIV/AIDS epidemic in China. UNAIDS (2012).

[17] Lu X, Zhao C, Wang W, Nie C, Zhang Y, Zhao H, et al HIV-1 genetic diversity and its distribution characteristics among newly diagnosed HIV-1 individuals in Hebei province, China. AIDS Res Ther. 2016; 19: 13:3. 10.1186/s12981-015-0087-2 PMC4719688 26793263

[18] Zhao C, Xing H, Zhao H, Huang H, Liu Y, Ma P, et al Subtyping and sequence analysis of the C2-V3 region of gp120 genes among HIV-1 strains in Hebei province. Chin J Microbiol Immunol. 2005; 25(7):533–5.

[19] Zhao C, Zhao H, Li B, Bai G, Xing H, Lu X, et al Molecular epidemiological study on HIV infection among paid blood donors. Chinese Journal of Health Laboratory Technology. 2010; 120(12):3136–40.

[20] Lu X, Kang X, Chen S, Zhao H, Liu Y, Zhao C, et al HIV-1 genetic diversity and transmitted drug resistance among recently infected individuals at Men Who Have Sex with Men sentinel surveillance points in Hebei province, China. AIDS Res Hum Retroviruses. 2015;31(10):1038–1045. 10.1089/AID.2015.0160 26200883

[21] Wu Z, Sullivan S, Wang Y, Rotheram-Borus M, and Detels R. Evolution of China’s response to HIV/AIDS. Lancet. 2007; 369(9562):679–90. 10.1016/S0140-6736(07)60315-8 17321313 PMC7137740

[22] Li L, Lu X, Li H, Chen L, Wang Z, Liu Y, et al High genetic diversity of HIV-1 was found in men who have sex with men in Shijiazhuang, China. Infect Genet Evol. 2011; 11(6):1487–92. 10.1016/j.meegid.2011.05.017 21645646

[23] Li L, Liang S, Chen L, Liu W, Li H, Liu Y, et al Genetic characterization of 13 subtype CRF01_AE near full-length genomes in Guangxi, China. AIDS Res Hum Retroviruses. 2010; 26(6):699–704. 10.1089/aid.2010.0026 20528151

[24] de Oliveira T, Deforche K, Cassol S, Salminen M, Paraskevis D, Seebregts C, et al An automated genotyping system for analysis of HIV-1 and other microbial sequences. Bioinformatics. 2005;21(19):3797–800. 10.1093/bioinformatics/bti607 16076886

[25] Lu X, Liu Y, Zhao H, Zhang Y, Zhao C, Chen S, et al Recombinant patterns of nine novel HIV-1 recombinant strains identified in Hebei province, China. AIDS Res Hum Retroviruses.2016; 32(5):475–9. 10.1089/AID.2016.0005 26892835

[26] Robertson DL, Anderson JP, Bradac JA, Carr JK, Foley B, Funkhouser RK, et al HIV-1 nomenclature proposal. Science 2000; 288(5463):55–6. 10766634 10.1126/science.288.5463.55d

[27] Han X, Takebe Y, Zhang W, An M, Zhao B, Hu Q, et al A large-scale survey of CRF55_01B from Men-Who-Have-Sex- with-Men in China:implying the evolutionary history and public health impact. Sci Rep 2015; 15;5:18147. 10.1038/srep18147 PMC4678862 26667846

[28] Li L, Sun B, Zeng H, Sun Z, Sun G, Yang R. Relatively high prevalence of drug resistance among anti-retroviral-naive patients from Henan, Central China. AIDS Res Hum Retroviruses. 2014;; 30(2):160–4. 10.1089/AID.2013.0144 23800338 PMC3910477

[29] Li L, Sun G, Liang S, Li J, Li T, Wang Z, et al Different distribution of HIV-1 subtype and drug resistance were found among treatment naïve individuals in Henan, Guangxi, and Yunnan province of China. PLoS One. 2013; 8(10):e75777 10.1371/journal.pone.0075777 24098398 PMC3789720

[30] Wang H, Liang BY, Zhou B, Jiang JJ, Huang JG, Chen RF, et al Distribution of subtypes of pol gene in HIV-1 epidemic strains in Guangxi Zhuang Autonomous Region, 2010–2012. Zhonghua Yu Fang Yi Xue Za Zhi. 2016; 50(1):79–84. 10.3760/cma.j.issn.0253-9624.2016.01.014 26792508

[31] Li L, Chen L, Yang S, Li T, Li J, Liu Y, et al Recombination form and epidemiology of HIV-1 unique recombinant strains identified in Yunnan, China. PloS One. 2012; 7(10): e46777 10.1371/journal.pone.0046777 23056447 PMC3467292

[32] Chen M, Ma Y, Duan S, Xing H, Yao S, Su Y, et al Genetic diversity and drug resistance among newly diagnosed and antiretroviral treatment-naive HIV-infected individuals in western Yunnan:a hot area of viral recombination in China. BMC Infect Dis. 2012; 12: 382 10.1186/1471-2334-12-382 23270497 PMC3552723

[33] Langford SE, Ananworanich J, Cooper DA. Predictors of disease progression in HIV infection: a review. AIDS Res Ther. 2007; 4: 11 10.1186/1742-6405-4-11 17502001 PMC1887539

[34] Li X, Xue Y, Zhou L, Lin Y, Yu X, Wang X, et a1 Evidence that HIV-1 CRF0l AE is associated with low CD4+T cell count and CXCR4 co-receptor usage in recently infected young men who have sex with men(MSM) in shanghai, china. PloS One 2014; 9(2):e89462 10.1371/journal.pone.0089462 24586795 PMC3931781

[35] Li YJ, Han Y, Xie J, Gu L, Li W, Wang H, et a1 CRF0l AE subtype is associated with X4 tropism and fast HIV pmgression in Chinese patients infected through sexual transmission. AIDS. 2014; 28(4):52l–530. 10.1097/QAD.0000000000000125 24472744

[36] Liu HX, wang L, Qin QQ, Ding ZW, Wang N. Impact of delayed diagnoses bias on the estimation of AIDS incubation. Chin J Epidemiol. 2011; 32(9):892–895. 22340877

[37] Li X, Lu H, Cox C, Zhao Y, Xia D, Sun Y, et a1 Changing the landscape of the HIV epidemic among MSM in China: results from three consecutive respondent-driven sampling surveys from 2009 to 2011. Biomed Res Int. 2014; 2014: 563517 10.1155/2014/563517 24575408 PMC3918367

[38] Yang L, Yang C, Chen H, Ma Y, Mei J, Su Y, et al HIV incidence in MSM in Yunnan, 2008–2011. Mod Prev Med. 2015;42(1):137–139, 156.

[39] Yang C, Ma Y, Chen H, Su Y, Chen M, Dong L, et a1 Application of BED for HIV-1 incidence research in female sex workers(FSW) in Yunnan province. Zhong hua Yu Fang Yi Xue Za Zhi. 2015;49(1):70–1. 25876501

[40] Duan S, Shen S, Bulterys M, Jia Y, Yang Y, Xiang L, et a1 Estimation of HlV-l incidence among five focal populations in Dehong, Yunnan: a hard hit area along a major drug trafficking route. BMC Public Health. 2010; 10:180 10.1186/1471-2458-10-180 20374618 PMC2858119

[41] Xu J, Smith MK, Ding G, Chu J, Wang H, Li Q, et a1 Drug use and sexwork: competing risk factors for newly acquired HIV in Yunnan, China. PloS One. 2013;8(3):e59050 10.1371/journal.pone.0059050 23555616 PMC3610908

