# Supplementary figures and images for: HIV-1 molecular epidemiology among newly diagnosed HIV-1 individuals in Hebei, a low HIV prevalence province in China
Source: PLoS One. 2017 Feb 8;12(2):e0171481. doi: 10.1371/journal.pone.0171481 (PMC5298910; doi:10.1371/journal.pone.0171481)

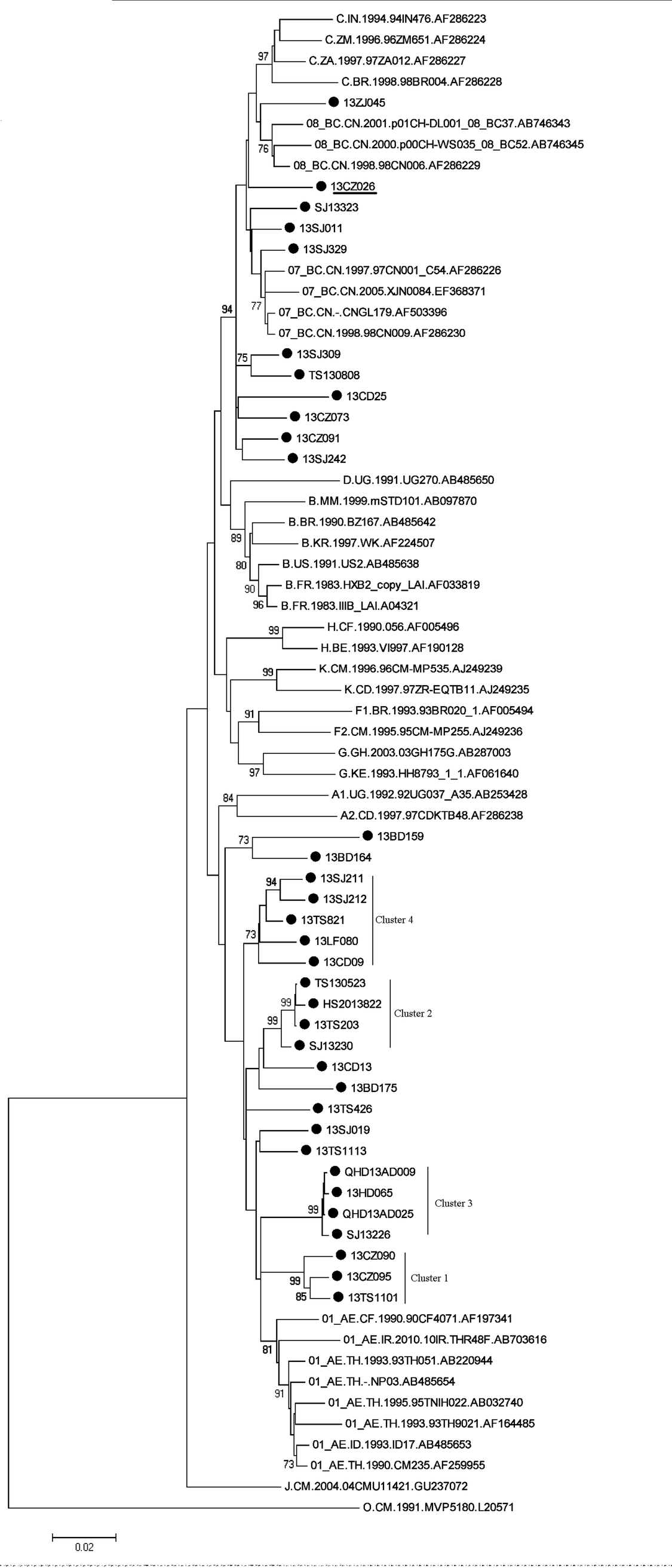

Supplement: S1 Fig — The reference gene sequences (A–D, F–H, J, K, O, CRF01_AE) were obtained from the HIV database (http://www.hiv.lanl.gov/content/index). The N-J phylogenetic tree was constructed using MEGA 5.0 with 1000 bootstrap replicates, based on Kimura 2-parameter Model. Bootstrap values ≥70% are shown in the tree. The scale length indicates 2% nucleotide sequence divergence. (TIF) [file pone.0171481.s001.tif]

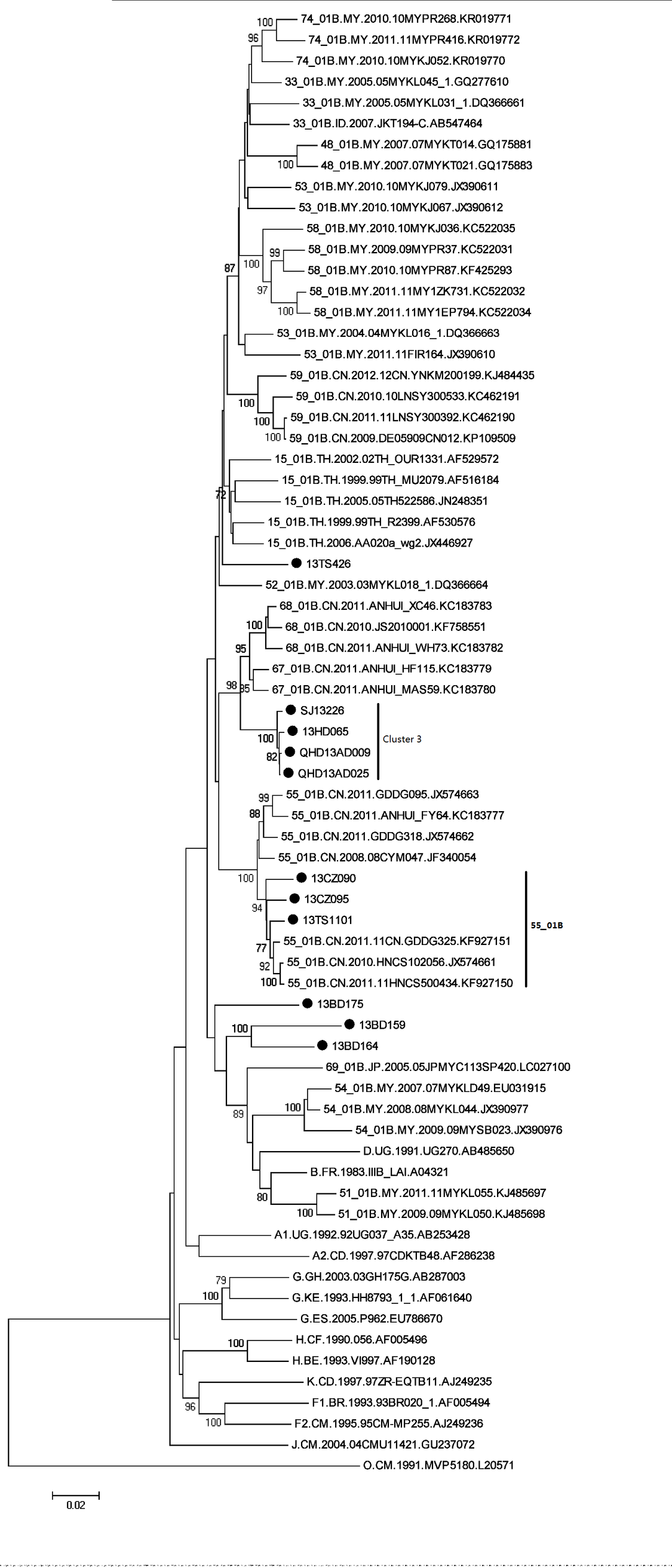

Supplement: S2 Fig — (TIF) [file pone.0171481.s002.tif]

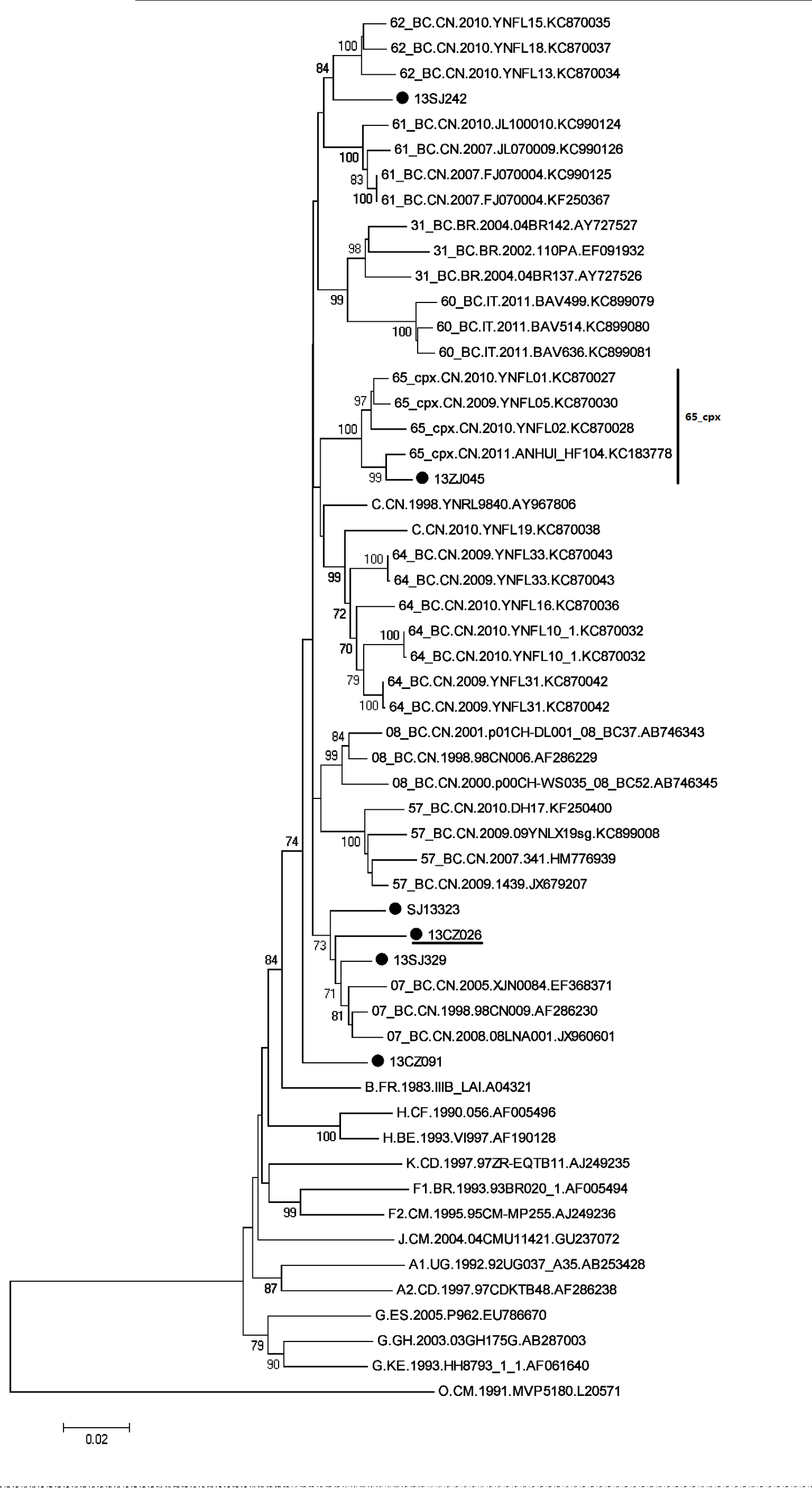

Supplement: S3 Fig — Of B/C recombinant strains, although jpMM-HIV analysis indicated that 13CZ026 (underlined) was subtype C, the N-J tree (Fig 2, S3), SimPlot 3.5.1 and RIP 3.0 (window size = 300) analysis confirmed that this recombinant strain was composed of subtype B and subtype C, and a small subtype B gene fragment was inserted into a subtype C backbone in the pol region. (TIF) [file pone.0171481.s003.tif]
