# Supplementary material for: HIV-1 molecular epidemiology among newly diagnosed HIV-1 individuals in Hebei, a low HIV prevalence province in China
Source: PLoS One. 2017 Feb 8;12(2):e0171481. doi: 10.1371/journal.pone.0171481 (PMC5298910; doi:10.1371/journal.pone.0171481)
Supplement: S1 Table — MSM: men who have sex with men. IDU: intravenous drug user. MTCT: mother to child transmission. (DOC) [file pone.0171481.s004.doc]

**S1 Table. Different risk groups in 11 prefectures of Hebei in 2013**

| Prefectures | | Subjects | MSM | Heterosexual | IDU | MTCT | Blood |
| --- | --- | --- | --- | --- | --- | --- | --- |
| Northern | Qinhuangdao | 35 (100) | 24 (68.6) | 10 (28.6) | 0 (0.0) | 1 (2.8) | 0 (0.0) |
|  | Tangshan | 81 (100) | 72 (88.9) | 9 (11.1) | 0 (0.0) | 0 (0.0) | 0 (0.0) |
|  | Zhangjiakou | 26 (100) | 18 (69.2) | 5 (19.2) | 3 (11.5) | 0 (0.0) | 0 (0.0) |
|  | Chengde | 18 (100) | 9 (50.0) | 9 (50.0) | 0 (0.0) | 0 (0.0) | 0 (0.0) |
|  | Subtotal | 160 (100) | 123 (76.9) | 33 (20.6) | 3 (1.9) | 1 (0.6) | 0 (0.0) |
| Central | Baoding | 97 (100) | 79 (81.4) | 15 (15.5) | 2 (2.1) | 1 (1.0) | 0 (0.0) |
|  | Cangzhou | 81 (100) | 51 (63.0) | 30 (37.0) | 0 (0.0) | 0 (0.0) | 0 (0.0) |
|  | Langfang | 47 (100) | 32 (68.1) | 10 (21.3) | 5 (10.6) | 0 (0.0) | 0 (0.0) |
|  | Shijiazhuang | 164 (100) | 84 (51.2) | 67 (40.9) | 11 (6.7) | 2 (1.2) | 0 (0.0) |
|  | Subtotal | 389 (100) | 246 (63.2) | 122 (31.4) | 18 (4.6) | 3 (0.8) | 0 (0.0) |
| Southern | Handan | 32 (100) | 23 (71.9) | 9 (28.1) | 0 (0.0) | 0 (0.0) | 0 (0.0) |
|  | Xingtai | 13 (100) | 6 (46.2) | 5 (38.5) | 0 (0.0) | 0 (0.0) | 2 (15.4) |
|  | Hengshui | 16 (100) | 13 (81.3) | 3 (18.7) | 0 (0.0) | 0 (0.0) | 0 (0.0) |
|  | Subtotal | 61 (100) | 42 (68.9) | 17 (27.9) | 0 (0.0) | 0 (0.0) | 2 (3.3) |
| Total |  | 610 (100) | 411 (67.4) | 172 (28.2) | 21 (3.4) | 4 (0.7) | 2 (0.3) |

MSM: men who have sex with men.

IDU: intravenous drug user.

MTCT: mother to child transmission.
