# Supplementary material for: HIV-1 molecular epidemiology among newly diagnosed HIV-1 individuals in Hebei, a low HIV prevalence province in China
Source: PLoS One. 2017 Feb 8;12(2):e0171481. doi: 10.1371/journal.pone.0171481 (PMC5298910; doi:10.1371/journal.pone.0171481)
Supplement: S2 Table — PIs:Protease inhibitors; NRTIs: Nucleoside reverse transcriptase inhibitors; NNRTIs: Non-nucleoside reverse transcriptase inhibitors. (DOC) [file pone.0171481.s005.doc]

**S2 Table. HIV-1 TDR-related mutations among 610 newly diagnosed-naïve individuals in 2013, Hebei**

| **Mutations** | **Prevalence** | | **Risk groups** | | **Genotypes** | | | | | | |
| --- | --- | --- | --- | --- | --- | --- | --- | --- | --- | --- | --- |
|  | N | % | MSM | H | 07_BC | B | 01_AE | A | URF | 55_01B | 08_BC |
| **PIs (N=34)** | |  |  |  |  |  |  |  |  |  |  |
| L10F | 2 | 0.3 | 2 |  |  |  | 2 |  |  |  |  |
| T74S | 2 | 0.3 | 1 | 1 | 1 |  |  |  | 1 |  |  |
| K20I | 10 | 1.6 | 3 | 7 |  | 1 | 8 |  | 1 |  |  |
| L33F | 2 | 0.3 | 2 |  |  |  | 2 |  |  |  |  |
| M46L | 10 | 1.6 | 8 | 2 | 1 |  | 9 |  |  |  |  |
| M46I | 10 | 1.6 | 7 | 3 |  |  | 10 |  |  |  |  |
| Q58E | 3 | 0.5 | 2 | 1 | 3 |  |  |  |  |  |  |
| V82F | 1 | 0.2 | 1 |  |  |  | 1 |  |  |  |  |
| L76LV | 2 | 0.3 | 1 | 1 | 2 |  |  |  |  |  |  |
| I84V | 1 | 0.2 |  | 1 | 1 |  |  |  |  |  |  |
| **NRTIs (N=14)** | | |  |  |  |  |  |  |  |  |  |
| K65EK | 3 | 0.5 | 2 | 1 |  | 1 | 2 |  |  |  |  |
| T69N | 4 | 0.7 | 2 | 2 |  |  | 3 |  | 1 |  |  |
| V75L | 1 | 0.2 | 1 |  |  |  | 1 |  |  |  |  |
| T215AT | 1 | 0.2 |  | 1 |  |  |  |  | 1 |  |  |
| T215D | 1 | 0.2 |  | 1 |  |  |  |  |  |  | 1 |
| L210W | 1 | 0.2 | 1 |  |  |  | 1 |  |  |  |  |
| K219E/Q/R | 3 | 0.5 | 3 |  | 1 |  | 1 |  | 1 |  |  |
| **NNRTIs (N=57)** | | |  |  |  |  |  |  |  |  |  |
| H221Y | 1 | 0.2 | 1 |  |  |  | 1 |  |  |  |  |
| K101E | 1 | 0.2 | 1 |  |  | 1 |  |  |  |  |  |
| K103N | 1 | 0.2 |  | 1 | 1 |  |  |  |  |  |  |
| V106A | 1 | 0.2 | 1 |  | 1 |  |  |  |  |  |  |
| V106M | 1 | 0.2 |  | 1 | 1 |  |  |  |  |  |  |
| V108IV | 3 | 0.5 | 2 | 1 | 2 | 1 |  |  |  |  |  |
| E138G | 1 | 0.2 | 1 |  |  | 1 |  |  |  |  |  |
| V179D/E/T | 41 | 6.7 | 27 | 14 | 3 | 3 | 30 |  | 1 | 3 | 1 |
| Y181C | 1 | 0.2 |  | 1 |  |  |  |  | 1 |  |  |
| G190E | 1 | 0.2 | 1 |  |  |  | 1 |  |  |  |  |
| F227FL | 1 | 0.2 | 1 |  | 1 |  |  |  |  |  |  |
| E138AE | 4 | 0.7 | 2 | 2 | 1 |  | 1 | 1 |  |  | 1 |
| N348I | 1 | 0.2 | 1 |  |  |  | 1 |  |  |  |  |

PIs:Protease inhibitors; NRTIs: Nucleoside reverse transcriptase inhibitors; NNRTIs: Non-nucleoside reverse transcriptase inhibitors
